# Experiences and Preferences for End-of-Life Care for Young Adults with Cancer and Their Informal Carers: A Narrative Synthesis

**DOI:** 10.1089/jayao.2016.0055

**Published:** 2017-06-01

**Authors:** Nothando Ngwenya, Charlotte Kenten, Louise Jones, Faith Gibson, Susie Pearce, Mary Flatley, Rachael Hough, L. Caroline Stirling, Rachel M. Taylor, Geoff Wong, Jeremy Whelan

**Affiliations:** ^1^ Cancer Clinical Trials Unit, University College Hospital, London, United Kingdom.; ^2^ Division of Psychiatry, Palliative Care Research Department, University College London, London, United Kingdom.; ^3^ Centre for Outcomes and Experiences Research in Children's Health, Great Ormond Street Hospital for Children NHS Foundation Trust, London, United Kingdom.; ^4^ School of Health Sciences, University of Surrey, London, United Kingdom.; ^5^ Department of Oncology, University College Hospital, London, United Kingdom.; ^6^ St. Joseph Hospice, London, United Kingdom.; ^7^ Children and Young Peoples Cancer Service, University College Hospital, London, United Kingdom.; ^8^ Camden, Islington ELiPSe and UCLH & HCA Palliative Care Service, Central and North West London NHS Trust, London, United Kingdom.; ^9^ Nuffield Department of Primary Care Health Sciences, University of Oxford, Oxford, United Kingdom.

**Keywords:** qualitative, end-of-life care, palliative care, systematic review, narrative synthesis

## Abstract

To review the qualitative literature on experiences of and preferences for end-of-life care of people with cancer aged 16–40 years (young adults) and their informal carers. A systematic review using narrative synthesis of qualitative studies using the 2006 UK Economic and Social Research Council research methods program guidance. Seven electronic bibliographic databases, two clinical trials databases, and three relevant theses databases were searched from January 2004 to October 2015. Eighteen articles were included from twelve countries. The selected studies included at least 5% of their patient sample within the age range 16–40 years. The studies were heterogeneous in their aims, focus, and sample, but described different aspects of end-of-life care for people with cancer. Positive experiences included facilitating adaptive coping and receiving palliative home care, while negative experiences were loss of “self” and nonfacilitative services and environment. Preferences included a family-centered approach to care, honest conversations about end of life, and facilitating normality. There is little evidence focused on the end-of-life needs of young adults. Analysis of reports including some young adults does not explore experience or preferences by age; therefore, it is difficult to identify age-specific issues clearly. From this review, we suggest that supportive interventions and education are needed to facilitate open and honest communication at an appropriate level with young people. Future research should focus on age-specific evidence about the end-of-life experiences and preferences for young adults with cancer and their informal carers.

## Introduction

In the United Kingdom, one quarter of deaths in those aged 16–40 (young adults) are cancer related, and in the European Union and United States, cancer is the leading disease-related cause of deaths in young adults.^1,2^ Overall survival rates for people with cancer aged 16–40 are improving, however, they are still less than those for children and older adults.^3,4^

While it has been acknowledged that the population aged 13–24 in the United Kingdom have specific needs that are catered for by specialist care, delivered, for example, in Teenage Cancer Units,^5^ there is a dearth of empirical research related to experiences and preferences for end-of-life care both for this age group and for younger adults with cancer aged 25–40.

End-of-life care is defined as “care that helps all those with advanced, progressive, and incurable illness to live as well as possible until they die.”^6^ Much care is treatment and cure directed, and there is little focus on understanding experiences when cure is not likely. Published literature has been confined to summaries of good practice, retrospective analysis of medical notes,^7^ or commentary and review articles.^8–11^ Research on older young adults (25–40 years) with cancer is limited, but has identified differences between age groups at end of life, including varying preferences for active treatment and greater symptom burden, or lower quality of life.^12–14^ In addition, experiences of those aged 25–40 are rarely considered separately from those who are much older.

Policy too has tended to overlook those aged 16–40 years. In the United Kingdom, the Department of Health report^15^ Better care: Better Lives makes no distinction between the needs of children, teenagers, and young adults, and focuses exclusively on children's palliative services. Similarly, the End-of-Life Care Strategy, Promoting High Quality Care for All Adults at the End of Life makes no specific reference to young adults.^6^ However, the World Health Organization has identified adolescents as one of the vulnerable groups whose palliative care needs are not being met and yet their death can be devastating with a lasting impact.^16^ Awareness of end-of-life care needs, experiences, and preferences of young adults with cancer may provide health professionals with evidence on how best to deliver optimal care for patients and their families.

In this systematic review using a narrative synthesis, we examine the extant research on the end-of-life care experiences and preferences of young adults and those of their informal carers, who are often family members. Our synthesis considers two age groups (16–24 and 25–40 years), and where possible, we highlight any findings that appear age specific. This age range reflects a wider study that the review was primarily conducted to inform. It reflects the distinction in the United Kingdom between Teenage and Young Adult cancer care for those aged 13–24 and those up to 40 years old in adult services. This review gives a comprehensive overview of studies that discuss any aspect of end-of-life care for people with cancer aged 16–40 years.

We aimed to answer the following questions:

(1) What are the experiences of and preferences for end-of-life care of young adults with cancer?
(2) What are experiences of and preferences for end-of-life care of the informal carers of young adults with cancer?
(3) What, if any, are the differences in experience and preferences between young adults aged 16–24 and 25–40?

## Methods

### Type of review

**FIG. 1.**
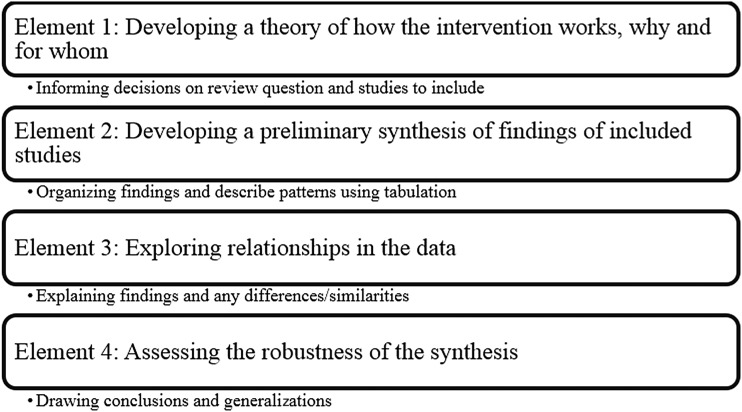
Elements in the process of a narrative Synthesis (from Popay et al., 2006).

We undertook a systematic review using narrative synthesis and applied a range of methods and techniques.^17^ According to Popay et al.,^17^ a narrative synthesis facilitates storytelling to bridge the gap between research, policy, and practice. It primarily takes a textual approach to “tell the story” of the evidence in the included studies. This had three discrete elements: textual summary, tabulation, and concept mapping (Fig. 1). This methodology enabled the aggregation of a substantive literature and description of factors that are identified relevant in end-of-life care for this population. Element 1 (Fig. 1) of the narrative synthesis method was not applied as we sought to aggregate literature on end-of-life care experiences and preferences, and therefore no theory was developed before the review.

### Literature search strategy

The databases AMED, BNI, CINAHL, EMBASE, HMIC, MEDLINE, and PsycINFO were initially searched from January 2004 (when the Improving supportive and palliative care for adults with cancer National Institute for Health and Care Excellence (NICE) guidelines were published in the United Kingdom) to June 2014 (by CK and NN) with updated searches run in October 2015 (Appendix 1 for a search strategy).

Four searches were constructed, conducted, and included; (1) people aged 16–24 with cancer, (2) informal carers of people aged 16–24 with cancer, (3) people aged 25–40 with cancer, and (4) informal carers of people aged 25–40 with cancer. According to the UK NICE guidelines, informal carers are defined as “lay people in a close supportive role who share in the illness experience of the patient and who undertake vital care work and emotion management,”^18^ and in practice were often family members. We combined MeSH terms and free text words for cancer, neoplasms, medical oncology, clinical oncology, palliative care, and end-of-life care with terms relating to patients and informal carers. These results were combined with MeSH terms and free text words for experiences and preferences (opinion, preference, perception, perspective). In addition, handsearching of reference lists, key journals, and gray literature was conducted, including controlledtrials, clinicaltrials, Opengrey, EThOSs, and Zetoc.

### Selection criteria

The search dates included articles published between January 2004 and October 2015. The population was young adults aged 16–40 with any cancer described as advanced, terminal, or incurable, and their informal carers. A narrative synthesis focuses on the textual to “tell a story” from the data in the included articles, and while it is possible to include statistical studies, for this review, only qualitative primary research studies reported in English, conducted in any country, were included. As this review was part of a larger project within a defined time frame, we did not have the resources for professional translation. We excluded studies if they used structured questionnaires as the sole method for data collection, or reported only quantitative data. Studies that did not elicit primary data from patients or carers were excluded.

### Quality assessment and data synthesis

The results from the searches were downloaded into bibliographic software (RefWorks) and deduplicated. A three-stage process was used to identify studies for inclusion.^19^ Two researchers (C.K. and N.N.) initially screened all remaining references for relevance based on title. Articles were then screened using abstracts for eligibility and relevance, and studies that did not use a qualitative design were excluded at this stage. Next, the full texts of potentially relevant studies were read and subject to an initial quality appraisal (by C.K. and N.N.) to remove ‘fatally flawed’ articles, that is, those of very poor quality following prompts from Dixon-Woods et al.^20^ (1) are the aims and objective of the research clearly stated; (2) is the research design clear and appropriate for the aims and objectives and (3) does the analysis reflect the methodology used in the study.

Remaining studies were assessed by the two researchers (C.K. and N.N.) and cross checked using the Critical Appraisal Skills Programme^21^ checklist for qualitative research with disagreements resolved by discussion with members of the core research team and tabulated (Table 1). Initially, a conservative approach on inclusion based on sample size was taken, excluding articles that did not adequately describe the sample age range. All authors of remaining articles were contacted through email and asked to confirm the age breakdown of their sample. As the paucity of young adult-specific studies or studies that included substantial proportions on young adults became apparent, it was decided to include studies if at least 5% of the sample was within the age range 16–40 to capture as much data as possible.

**Table 1.** Characteristics of Reviewed Articles

	*Authors*	*Location of study*	*Aim(s)*	*% of patients in AYA age range*	*Age group patients*	*Study design/data collection method*
Articles from studies with family members of patients aged 16–24	Barling et al.^29^	Australia	Describe the reality of hospitalization in the experience that accompanies the stages of diagnosis, treatment, dying, and death of an AYA from the perspective of informal carers.	87.5%	14 pts. aged 16–23	Open-ended interviews
	Cataudella and Zelcer^32^	Canada	Explore the psychological experiences of children with brain tumors at the end of life from parental perspectives	16.66%	4 patients aged 16–19	Semistructured focus group interviews
	Gaab et al.^34^	New Zealand	Describe caregiver's experiences of talking about their children's prognosis	21.05%	4 pts. aged 16–18	Semistructured interviews
	Montel et al.^42^	France	Investigate the place of death of adolescents and young adults and factors influencing the choice of place of death	50%	19 aged 16–24	Qualitative interview study
Articles from studies with patients aged 16–24	Dahlin and Heiwe^33^	Sweden	Elicit perceptions and experiences of physical therapeutic interventions from patients in palliative cancer care	16–24 = 5.88%; 25–40 = 5.88%	1 16–24; 1 25–40	Semistructured interviews
	Doumit et al.^25^	Lebanon	Uncover the lived experience of Lebanese oncology patients receiving palliative care	10%	1 pt. aged 21	In-depth semistructured interviews with observation field notes
	Williams^40^	United States of America	Describe the experience of existential suffering among low socioeconomic (SES) patients dying from cancer	up to 24 = 3.03%; 25–40 = 30.30%	1 pt. = 24; 10pts 25–40	In-depth open-ended interviews
Articles from studies with patients aged 25–40 years	Almack et al.^35^	United Kingdom	Explore if, how, and when advance care planning conversations were facilitated and documented	(based on 9 pts. with cancer) 11.1%	1 pt. aged 33	Exploratory case study using interviews
	Brom et al.^37^	Netherlands	Explore cancer patients' preferences and the reasons for patients' preferred role in treatment decision-making at the end of life.	11%	3 pts. under 35 years and 3pts. aged 36–50	In-depth interviews
	Hoff et al.^45^	Sweden	Investigate patients' views of information during the trajectory of their disease and the different reactions among patients	8.33%	1 pt. under 40- no specific age given	Recurrent semistructured interviews
	Milberg et al.^28^	Sweden	Explore palliative home care as a secure base based on patients and family members' experiences	8.33%	1 pt. aged 35	Interviews
Articles from studies with patients aged 25–40 years	Nedjat-Haiem^36^	United States of America	Explore perceptions of the barriers to engaging in EOL decision-making discussions specifically among low-income Latinos living with an advanced life-threatening cancer condition	22.22%	2 pts. (35, 39)	In-depth semistructured interviews, patient observations
	Nilmanat et al.^23^	Thailand	Describe the experience of living with suffering for patients with terminal advanced cancer in Thailand	6.66%	1 pt. 30	Longitudinal study using series interviews (based on the health and willingness of participant) and participant observations, field notes
	Philip et al.^31^	Australia	Understand the lived experiences of patients and perceptions of current health services	10%	1 pt. aged 40	In-depth interviews
	Rydahl-Hansen^24^	Denmark	Describe the experienced suffering among hospitalized patients with incurable cancer	8.33%	1 pt. was 40 years old	Series of semistructured interviews, and observations
	Sand et al.^27^	Norway	Explore the experience of using medicines for patients with far-advanced cancer with a short life expectancy	6.66%	1 pt. aged 39	Interviews
	Volker and Wu.^26^	United States of America	Explore the meaning of control and control preferences in racially and ethnically diverse patients with cancer	15%	3 pts. (40, 39 & 34)	In-depth interviews
	Worth et al.^30^	United Kingdom	Explore experiences and perceptions of out-of-hours care of patients with advanced cancer	6.25%	2 pt. under 40 (aged 30–40)	Individual interviews with patients and focus groups with patients and carers

In relation to the process of synthesis, common concepts were identified in the articles, which describe the experiences and preferences of end-of-life care (Table 2).

**Table 2.** Tabulation of Articles with Emerging Concepts of Experiences and Preferences

*Study*	*Sample*	*Research findings*	*Concepts for experience*	*Concepts for preference*
Almack et al.^35^	18 cases made of patients, relatives, and healthcare professionals; 9 patients with cancer, 4 with heart failure, 2 with multiple sclerosis, and 3 with stroke and comorbidities	Reluctance to discuss end-of-life issues; assumption that healthcare professionals will initiate the conversations; professionals hesitant to initiate the conversations as they perceived this as taking away the patient's hope	No end-of-life discussions	Healthcare professionals to initiate end-of-life discussions
Barling et al.^29^	26 informal carers who had experienced the death of an adolescent or young adult with cancer	Negative impact of hospitalization; importance of place and space; treatment environment not appropriate for adolescents and young adults; hospital environment not conducive to healing; young adults do not fit within system; hospitalization is like a death sentence	Confinement of hospitalization; space not conducive to healing; not fitting in	Age-appropriate environment
Brom et al.^37^	28 advanced care patients	Desire for doctor to play a role in decision-making process; considered doctor's expertise, knowledge, and experience in treatment decisions; other patients want to participate in decisions; other patients preferred the physicians to play a decisive role; others wanted to maintain control by having a more decisive role; role in decision-making changed with the fluctuating status of illness and aim of the treatment; want more active role when disease progressed	Involvement in decision-making	Doctors to share expert voice; desire to maintain control
Cataudella and Zelcer^32^	24 bereaved parents of children diagnosed at less than 18 years of age with a brain tumor	Emotional and cognitive changes; awareness of death; desire to be treated as normal; to remain connected with others; and post-traumatic growth	Emotional changes; cognitive changes;	Normalcy; connection with others
Dahlin and Heiwe^33^	17 patients with advanced cancer	Physical therapy clear and satisfactory; therapists viewed as good listeners; may feel a burden; time-limited improvement; lack of information; independence is important for patients; preserve autonomy	Confidence from therapy	Independence; control; assistance to gain motivation
Doumit et al.^25^	10 patients. Lived experience of cancer between 2–21 years	Distress from being dependent; dislike of pity; worry for family and family worry; reliance on God and divinity; dislike of hospital; need to be productive; fear of pain; need to communicate	Distress; worry for others; pain	Rely on God; being productive
Gaab et al.^34^	19 primary caregivers of a child receiving palliative care aged 3–18 years	Support from family both physically and emotionally; support with decision-making aspects; feelings of regret and blame; parental roles; disability discrimination; use of the internet to seek and offer help	Support from family/friends; feelings of regret; blame; disability discrimination	Use of internet; need for information
Hoff et al.^45^	12 patients; 7 with malignant hematological disease, 5 with nonoperable lung cancer	Well informed initially; less information with disease progression; information dependent and accepting of the news; information dependent but denying; medically informed and accepting and medically informed but denying	Less information with disease progression; information dependent; acceptance; denial; medically informed	Need information on bad news
Milberg et al.^28^	12 patients, and 14 unconnected informal carers	Palliative home care as a secure based; having a sense of control; experiencing inner peace; having trust in the staff; being recognized as an individual; family being relieved of the burden of responsibility being informed, feeling welcome; and the ability to continue with everyday life at home; loss of self; loneliness; death anxiety	Security; death anxiety	Security; a sense of control
Montel et al.^42^	38 parents of adolescents and young adults aged 15–25 who died at Institut Curie	Children aware of imminent death; obstacles to talking about death; regrets of not talking about dying; parents concerned about child's place of death and home care; need for psychological support; none of the parents used the bereavement services after the child's death	Representation of death; awareness of death; not talking about dying; feelings of regret	Need for information; psychological support; bereavement support
Philip et al.^31^	10 patients with primary malignant glioma grade 3–4	Loss of self; feelings of vulnerability; fear of being a burden to others; feelings of loneliness and isolation; lack of openness by healthcare professionals; all about waiting and uncertainty	Uncertainty; lack of openness from professionals; being a burden	
Rydahl-Hansen^24^	12 patients with incurable cancer with a Folstein's Mini-Mental State Examination score of 24 points or more, born in Denmark by Danish parents	Suffering from increasing powerlessness; loneliness and isolation; struggle to maintain or regain control	Increasing powerlessness; loneliness; isolation; control	Maintain control
Sand et al.^27^	15 patients with advanced incurable cancer and a short life expectancy	Fear of losing control; worry and confusion over medication; fear of becoming addicted to medication; gain control by not taking medication; patients wanted to self-manage rather than comply	Loss of control	Self-management; shared decision-making with professionals
Nedjat-Haiem^36^	9 triads of patient, informal carer, and provider, and one patient–provider pair	Lacking understanding of severity of illness; hope to be cured; end-of-life discussions deferred by professionals; patients informed of progression at time of crisis; gaps in end-of-life communication; omission of vital information	Hope; severity of illness; gaps in communication	Open and honest communication; information on prognosis
Nilmanat et al.^23^	15 patients with life expectancy of less than 6 months	Living with suffering; distress caused by physical symptoms; alienation; sense of worthlessness, sense of being a burden to others; a desire to hasten death	Living with suffering; being a burden; alienation	Desire to hasten death
Volker and Wu ^26^	20 patients with advanced cancer	Wanting control; higher power exists with overall control of cancer and death	Control; belief in higher power	Control of everyday life; involvement in treatment decisions
Worth et al.^30^	32 patients with advanced cancer; ranged from those receiving palliative treatment to the terminally ill	Reluctance to call out of hours services; anxiety about the importance of their need; did not want to bother the doctor; perceived triage as a blockade to access to care; received effective planning; empathic responses from staff	Effective planning; triage as blockade to care	Effective planning; confidence to access care; empathic professionals
Williams^40^	33 outpatients from an oncology clinic of a public hospital aged 20–70+	Terminal illness career; changes of inner self; changes in outer interactions; Emotional labor; managing own and others feelings	Emotional labor; external physical changes	Normalcy; maintain interactions; social networks

For element 2 of the process of a narrative synthesis, we used both tabulation and textual description through thematic approach as a preliminary synthesis (Table 1).^22^ For element 3, concepts were identified in the included studies, which describe end-of-life care experiences and preferences of young adults with cancer and their carers (Table 2).

**FIG. 3.**
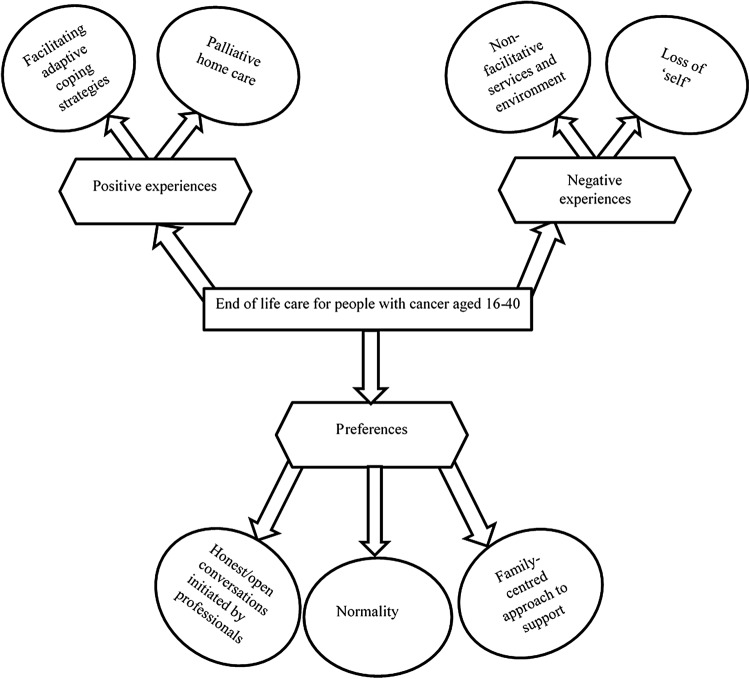
Key concepts in the experiences and preferences of people with cancer aged 16–40 when cure is not likely.

Conceptual mapping was used to explore the connections and relationships within and between the studies in the review. Figure 3 is a diagrammatic representation of the key concepts relevant to the review question. Gough's weight of evidence framework was used to assess quality, appropriateness, and relevance of the study to the review question. The scores of “low”, “medium.” or “high” are given for each weight of evidence as shown in Table 3. Of the eighteen studies included in the review, four received an overall “high” weight, eleven classified as “medium,” and three were given an overall weight of “low”. This quality assessment does not have much impact on the synthesis of the review as the inclusion criteria were used more reliably to either exclude or include studies.

**Table 3.** Weighting of Studies by Quality According to Four Criteria [Strength of Evidence (EPPI Approach)]

*Study*	*Weight A–methodological soundness*	*Weight B–appropriateness*	*Weight C–relevance*	*Weight D–overall weight*
Almack et al.^35^	Medium	High	High	High
Barling et al.^29^	Medium	High	High	High
Brom et al.^37^	High	Medium	High	High
Cataudella and Zelcer^32^	High	High	High	High
Dahlin and Heiwe^33^	Medium	Medium	Low	Low
Doumit et al.^25^	Low	Low	Medium	Medium
Gaab et al.^34^	High	Medium	High	High
Hoff et al.^45^	Low	High	High	High
Milberg et al.^28^	Medium	Medium	High	High
Montel et al.^42^	Medium	High	High	High
Philip et al.^31^	Medium	Medium	Medium	Medium
Rydahl-Hansen^24^	Low	Medium	Low	Low
Sand et al.^27^	Medium	Low	Low	Low
Nedjat-Haiem^36^	High	High	High	High
Nilmanat et al.^23^	Low	Medium	Medium	Medium
Volker and Wu^26^	High	High	High	High
Worth et al.^30^	Medium	Low	Medium	Medium
Williams^40^	Low	Medium	Medium	Medium

## Results

### Searches

**FIG. 2.**
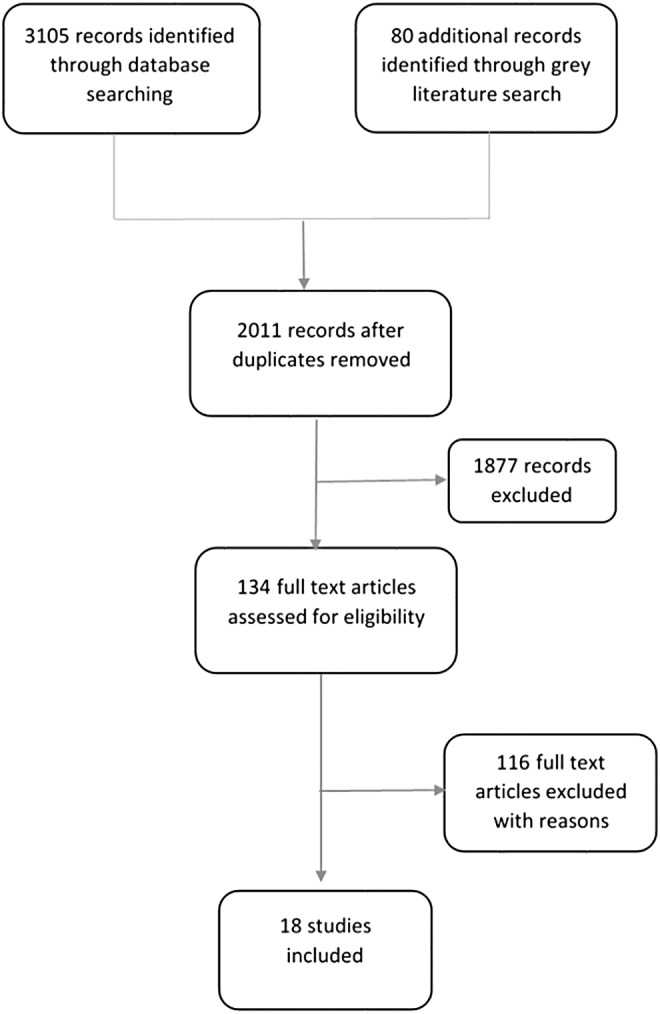
PRISMA Flowchart of literature search.

The initial search between January 2004 and June 2014 yielded 873 citations that related to patients and 773 to informal carers, which gave a total of 837 references after duplicates were removed. Forty two records were identified in the gray literature. An updated search was run between January 2014 and October 2015. After screening and quality assessment, two further studies were included. The final number of included articles was 18 (Fig. 2).

### Negative experiences

Although young people with terminal cancer experience many changes, the feeling of a loss of “self” is one that permeates all others and impacts on many other psychosocial functioning such as control, feelings of worthlessness, autonomy, and increased dependency.^23,24^ This was mostly reported in the studies with patients aged 25–40 years who are likely to have lives independent of their parents. Western society normally perceives cancer as a disease for older people. A cancer diagnosis is unexpected, and a terminal prognosis is a shock and even more unexpected for young people. It often enforces role changes in a young person's life making them unproductive.^25^

This disruption increases the desire that people have to retain control of their everyday lives up to the end of life.^26^ There are many aspects of having terminal cancer that have an impact on control for patients, including the anxiety associated with medication they take for their symptoms and how this could potentially contribute to losing a sense of self.^27^ For this younger population, who are in a life stage of developing independence, having cancer compromised their concept of ‘self’ and reduced their independence and control they have over their lives, causing distress and leading to dependence on others.^25^ This lack of control over symptoms and everyday life gave patients a sense of insecurity.^28^

Patients felt that a hospital stay represented a deterioration in their disease progression or perceived it as a place to die.^25^ The experience of interaction with “out-of-hours” services for those aged 25–40 was related to inability to access care due to their hesitancy in contacting out of hours palliative care services, and uncertainty of their needs and nonfacilitative services.^30^ End-of-life patients felt that other needs involving existential concerns were not addressed by the current medical system and carers were not linked to appropriate support.^31^

Reports from informal carers of dying patients, who were usually family members, described how receiving care at a hospital made cancer more of a reality for them and for the patient and that, neither the pediatric nor adult systems of care or spaces were appropriate for end-of-life care and there was no specific system of care available for these young adults.^29^

### Positive experiences

Palliative home care offered a secure base and hope at a time when patients and family members were feeling vulnerable, and increased their trust in the professionals.^28^ Patients felt they were recognized and treated as an individual with palliative home care, gaining a sense of control, while informal carers welcomed the relief in responsibility of caring for the patient.^28^

Post-traumatic growth or the positive psychological change was identified as helpful for adolescents to develop resilience.^32^ The awareness of a limited time also prompted young people to try and accomplish many things in the time they had left, thus developing an adaptive attitude as a coping strategy.^32^ These adaptive coping strategies helped the patients and their parents to deal with the anticipatory grief and the imminent death of the patient. Physical therapeutic interventions were perceived to enable independence and empowered patients with confidence, security, and hope at a time of vulnerability.^33^

### Preferences

A key process characterizing adolescents and young adults' preference when facing terminal cancer is maintaining normality. Patients wanted to be treated as normally as possible, thereby helping them to stay connected with their social networks and continuing with daily activities.^23,32,34^ Being treated like their healthy peers normalized their situation, decreasing discrimination experienced and increasing their coping.^34^ Continuing with everyday life and maintaining precancer activities helped them to cope and represent their sense of normality as their prognosis deviates from what is perceived as a normal life course. This normalization process helped them maintain their sense of “self” and identity.^28^

When it comes to communication preferences at end-of-life, patients and families reported that advance care planning was difficult to initiate.^31,35,36^ Although guidance and professionals acknowledge that it is best to open these conversations as early as possible, in practice this does not happen, with professionals, patients, and family members all reticent to engage in these difficult conversations that had implications for planning for the future.^24,28,31,35^ Patients wanted professionals to play an active role in these discussions and treatment decisions due to their expertise.^37^ The need for open communication helped individuals accept the reality of their prognosis, alleviating anxieties and uncertainties associated with not knowing.^25^ Having an open communication can also alleviate the feeling of powerlessness that patients sometimes experience.^33^

#### Families and informal carers

The importance of informal carers, who were often family members, is evident in relation to good end-of-life care in the home setting. Individuals exist within a society and therefore should be considered in the context of “relational self”. “Relational self” refers to how individuals are interconnected within society and significant others have an impact and influence on life experiences.^38,39^ The process of dying is experienced by a family within the social context.^40^ Families play a central role in palliative care and need support to do this effectively. Families want to be involved in the decision-making processes as it impacts on their role as carers and would like to feel welcomed by the professionals.^28,34^

Parents often felt it was their responsibility to know and interpret their child's voice, which they found stressful and challenging especially when it contradicted their view as a parent and they struggled between preserving their child's life and letting go.^41^ Primary carers, who were usually the parents, experienced many negative emotions such as regret, distress, and guilt. This had an impact on their psychological wellbeing and thus required a supportive response from services on a family and individual level to improve palliative care for patients.^34^ Bereaved parents of those aged 16–24 regretted not having the opportunity to talk about end-of-life care with their son or daughter and expressed how this could have helped prepare for their child's death and the bereavement process.^32,42^

## Discussion

There is research on end-of-life care, but very little is specifically focused on the experiences and preferences of young adults within our included age range of 16–40 years. However, the themes identified are relevant to young people and are consistent with their developmental stage. The concept of having a short life can cause distress coupled with uncertainty of the future and a loss of self.^23,24,40^ Having a terminal diagnosis changes a patient's perspectives and expectations of life, forcing them to take on a new role and identity in an attempt to normalize their life and continue life with this new status. The new status, however, comes with many internal psychological processes, including changes emotionally and cognitively. Not only do the young people experience a loss of self but also anticipated loss of their future and of interpersonal relationships.^32^

As highlighted within this review, young adults experienced feelings of loneliness and isolation and have an increased desire to be treated the same as their healthy peers, and to maintain some interpersonal connection.^34^ This can sometimes be difficult as their disease progresses causing disabilities that make them either housebound or hospital bound. Assistance from professionals with adaptive coping was identified as a preferred way of helping young people. Another concept we identified is the importance of the environment in which young adults are treated and cared for.^29^ Family members who were informal carers reported the impact that the location of care had on young people, which can either facilitate or impede the healing process. Healing process in this context does not refer to cure, however, the mechanisms involved in achieving emotional wellness.

Hospital as a place of care for most young adults was not conducive to healing and reported as a confined space, which was described as a “nightmare”.^25,29^ This raises the need for a greater understanding of the care needs of young adults with cancer as they will all inevitably require treatment from the hospital in their journey.

Palliative home care was identified as a service that equipped patients and families with hope and a secure base. Evidence has shown how caring for someone at home with cancer can be burdensome.^43,44^ Palliative home care afforded family members the relief from responsibility as they had a professional to share the burden of caring for the patient at home, giving them support and a sense of security.^28^ For patients, palliative home care helped them maintain their sense of “self” and felt that they were treated as an individual within the secure environment of their home.

Communication is still a major concern for young adults in relation to end-of-life care conversations. In some instances, the patient's voice may not be heard and it may be difficult to integrate both the patients' and families' desires. There is a lack of direct discussion about end-of-life care, and information is not tailored to the interest of young adults, which may have an impact on treatment decisions and planning for the future with less information disclosed with disease progression.^35,37,45^

Healthcare professionals and family members in their role as informal carers seem to be uncomfortable discussing death, which has a direct impact on patients, and practical implications for advance care planning.^26^ Patients have an expectation for professionals to initiate conversations as they have the expertise, knowledge, and experience.^31,36^ Although one article reported hesitancy about accessing out-of-hours services,^29^ other articles discuss the nonfacilitative experiences of services, which hindered satisfaction with care.

### Strengths and limitations

The database of the search strategy used in this review was sensitive and effective, but broad as well. This means that there was a breadth of studies included in the review eliminating the possibility of missing out any relevant studies. Inclusion of all the studies, however, means some of the studies were of low quality, and although over half of the studies incorporated into the synthesis had only 1 or 2 patients, the sample size limits the strength of the findings. We selected a very permissive threshold of 5% of patients within the age range of 16–40 for study inclusion, reflecting the dearth of research that is specific to the end-of-life care needs of young adults with cancer.

The findings identify aspects that may be generic to the population of people living with a terminal illness, but are accentuated with the younger population. The heterogeneity of the studies made it difficult to conduct an effective quality appraisal. The review only included English language studies, which is another limitation as this may have led to the omission of relevant articles.

### Implications for policy and practice

Although all people living with a terminal illness have similar concerns at the end of life, considering the individual development of the patient and their functioning at that stage of life is important in implementing appropriate support for patients and their families. This review reports how young adults may feel that the hospital environment does not facilitate healing, which raises the need to address this specific issue. Healthcare systems must place a greater emphasis on the care and treatment environment as this has a significant impact on young people's overall experience on the care they receive. Although patients' concerns may be highly individual, professionals need to acknowledge the needs of this specific population and that of their family to deliver care that is appropriate to their circumstances.

This review has highlighted some issues of concern and needs that young adults may have, including the uncertainty associated with the lack of end-of-life care conversations. This is a concern that needs to be addressed at population level, making palliative care a public health issue. Most research so far has neglected the influence of circumstantial age, and care systems do not recognize this impact on young people.

The focus of concern for service providers should extend not only to considerations of chronological age but also to circumstantial age, the particular patient's life cycle, and tasks they have at that stage to effectively engage with the patient and provide support that is relevant. Hospital care models should provide activities or support that alleviates the feelings of confinement and isolation that young adults experience and provide a more creative landscape. Policies need to recognize the work that informal carers, who are often family members, contribute within the healthcare system and provide support both before and into bereavement. A family-centered approach to care may help to engage with family members, supporting them in the important role they play in end-of-life care.

## Appendix

**Appendix 1.** Example of a Search Strategy

1. BNI; palliative.ti,ab;
2. BNI; palliati^*^.ti,ab;
3. BNI; (“end of life” OR “end of life care”).ti,ab;
4. BNI; dying.ti,ab;
5. BNI; “advance care planning”.ti,ab;
6. BNI; 1 or 2 or 3 or 4 or 5;
7. BNI; exp CANCER/;
8. BNI; neoplasm.ti,ab;
9. BNI; oncology.ti,ab;
10. BNI; ((haematology OR hematology)).ti,ab;
11. BNI; cancer.ti,ab;
12. BNI; 7 or 8 or 9 or 10 or 11;
13. BNI; view^*^.ti,ab;
14. BNI; satisfaction^*^.ti,ab;
15. BNI; preference^*^.ti,ab;
16. BNI; opinion^*^.ti,ab;
17. BNI; perspective^*^.ti,ab;
18. BNI; concern^*^.ti,ab;
19. BNI; issue^*^.ti,ab;
20. BNI; experience^*^.ti,ab;
21. BNI; journey^*^.ti,ab;
22. BNI; pathway^*^.ti,ab;
23. BNI; ((worry OR worries)).ti,ab;
24. BNI; attitude^*^.ti,ab;
25. BNI; exp DEATH: ATTITUDES/OR exp PATIENTS: ATTITUDES AND PERCEPTIONS/; 6215 results
26. BNI; patients.ti,ab;
27. BNI; exp PATIENTS/;
28. BNI; 13 or 14 or 15 or 16 or 17 or 18 or 19 or 20 or 21 or 22 or 23 or 24 or 25;
29. BNI; 26 or 27;
30. BNI; 6 and 12 and 28 and 29;
31. BNI; 30 [Limit to: Publication Year 2004–2014]; [Limit to: English];
